# Does Urinary Incontinence and Mode of Delivery Affect Postpartum Depression? A Nationwide Population-Based Cohort Study in Korea

**DOI:** 10.3390/ijerph18020437

**Published:** 2021-01-08

**Authors:** Jin Young Nam, Eun-Cheol Park, Eun Cho

**Affiliations:** 1 Department of Healthcare Management, Eulji University, Sungnam-si 13135, Gyeonggi-do, Korea; jynam@eulji.ac.kr; 2 Department of Preventive Medicine, Yonsei University College of Medicine, Seoul 03722, Korea; ecpark@yonsei.ac.kr; 3 College of Pharmacy, Sookmyung Women’s University, Seoul 04310, Korea

**Keywords:** urinary incontinence, postpartum depression, mode of delivery, cohort study

## Abstract

We investigated the association between urinary incontinence and postpartum depression. Data were extracted from the Korean National Health Insurance Service-National Sample Cohort and included women who delivered between 2004 and 2013. Postpartum depression was determined using diagnostic codes during the six-month postpartum period. Urinary incontinence was identified as having a prescription of incontinence drugs or a diagnosis. Cox proportional hazard models were used to calculate adjusted hazard ratios. Of the 83,066 women, 5393 (6.49%) had urinary incontinence and 691 (0.83%) had postpartum depression. Postpartum depression was higher among women with urinary incontinence, aged 15–19 years, ≥40 years old, the lowest income level, and who underwent cesarean section delivery. In the combined analysis, women with urinary incontinence and cesarean section had an approximately three times higher risk of postpartum depression compared with those without urinary incontinence and with spontaneous delivery. Women without urinary incontinence and cesarean section, and those with urinary incontinence and spontaneous delivery were at higher risk of postpartum depression compared with the reference group. Urinary incontinence and cesarean section delivery were significantly associated with postpartum depression during the first six months after childbirth. Therefore, further research should be conducted to evaluate whether urinary incontinence contributes to postpartum depression.

## 1. Introduction

Postpartum depression (PPD) is a common mental health problem among reproductive-aged women, occurring in approximately 13–19% of women during the first six months after delivery [1]. PPD has adverse consequences on maternal and infant health, mother-infant bonding, and infant development [2]. Even though PPD is a serious problem, it is only diagnosed and treated in around 1% of mothers in Korea [3] because of adverse perceptions of mental disorders and the fear of social stigmatization often leading to concealment of symptoms [4]. Previous studies have demonstrated that risk factors for PPD include lower socioeconomic status and social support, childcare stress, a personal or family history of depression, cesarean section delivery, lack of breastfeeding, and adverse physical symptoms [1,5,6,7]. In a previous study, triggering of PPD was potentially explained as reproductive hormonal changes, especially reduction in estrogen and dysregulation of postsynaptic serotonin function, leading to the onset of clinical depression in a vulnerable subgroup of women [1,8]. However, this is not sufficient because of the presence of a number of other potential contributing factors of depression. In the general population, it is difficult to identify which patients will develop depression, as well as the timing and relationship between risk factors [9]. In contrast, postpartum depression is relatively well-defined in terms of the periods of onset. Therefore, it is important to determine sensitive markers to predict preventable factors for postpartum depression in women.

Urinary incontinence (UI) is one of the most common conditions among postpartum women, occurring in 5–36% [10]. UI can lead to adverse effects on health-related quality of life levels among postpartum women [11]. Previous studies have shown that risk factors such as body mass index (BMI), parity, perineotomy, prolonged second stage of delivery, forceps delivery, and advanced maternal age at first childbirth were related to increased rates of post-pregnancy UI [10,12]. Spontaneous vaginal (SV) delivery was associated with a higher risk of UI post-delivery compared with cesarean section (CS) delivery [10,13]. However, most studies have only generally estimated the effects of UI on overall physical health with specific effects of UI on mental health after childbirth currently not adequately examined [14,15]. Although there have been a few studies on the relationship between urge UI, overactive bladder syndrome, and PPD, these were cross-sectional, clinic-based studies with few participants and a selection or recall bias [9,16], or they were prospective cohort study designs that included only delivered singletons [5]. Another study showed that a diagnosis of depression could increase the risk of UI, but this study’s average age was 59.3 years and it was not conducted on postpartum women [17].

There is much evidence of the association between the mode of delivery and PPD. Several epidemiological studies have shown that there is a relationship between an increased risk of PPD and vacuum extraction delivery [18], emergency cesarean section, or elective cesarean section [19], although some studies state that the impact of the mode of delivery may be an indirect one. Regardless, there remains limited evidence of a relationship between UI and mode of delivery on PPD. Therefore, there is a need to evaluate these associations using a longitudinal population-based cohort study design.

Hence, this study investigated the association between UI and PPD six months after childbirth using a longitudinal population-based cohort design. Moreover, we analyzed the effect of other risk factors such as the association of mode of delivery on PPD.

## 2. Materials and Methods

### 2.1. Study Participants and Data Collection 

The study population was obtained from the Korean National Health Insurance Service-National Sample Cohort (NHIS-NSC) data, which consisted of a nationwide representative (*n* = 1,025,340, 2.5%) cohort from the Korean population in 2002. The cohort was made through random sampling based on stratification by age, sex, residence area, type of health insurance, household income decile, and total medical costs for individuals. It tracked all information about the clinical characteristics of the cohort between 2002 and 2013. NHIS-NSC was recorded as unique de-identified patient numbers containing information regarding age, sex, type of insurance, diagnosis codes according to the International Classification of Diseases, 10th revision (ICD-10), procedure codes, prescribed drugs, and total medical costs. Additionally, the unique de-identified numbers were connected to mortality data from the Korean National Statistic Office.

We excluded women who had a history of depression before and during pregnancy. Those who delivered between 2002 and 2003 (*n* = 16,603) were also excluded because their history of depression before and during pregnancy could not be determined using the database. In total, the final cohort included 83,066 women who delivered between 2004 and 2013. This study adhered to the tenets of the Declaration of Helsinki and was reviewed and approved by the ethics board of Sookmyung Women’s University (SMWU-1808-HR-076).

### 2.2. Postpartum Depression (PPD) and Follow-Up

PPD was the primary outcome variable of this study. PPD was defined as an outpatient or inpatient visit at least once during the first six months postpartum period containing a new diagnosis of any of the following depressive disorders: bipolar affective disorder (ICD-10: F31), depressive episodes (F32), recurrent depressive disorder (F33), persistent mood disorders (F34); other mood disorders (F38); unspecified mood disorder (F39); mixed anxiety and depressive disorder (F41), or mental disorders associated with the puerperium (F53). We searched for occurrences of PPD using the above criteria in the time period between 1 January 2004 and 31 December 2013 on the NHIS-NSC database. 

### 2.3. Urinary Incontinence (UI)

We assessed UI based on diagnostic codes and prescription drug records. The related diagnostic codes searched for included: urge UI (N3940), overactive bladder syndrome (N328), stress UI (N393), mixed (N3941), other (N3948) incontinence, or unspecified UI (R32). UI was also diagnosed if patients had at least one or more of the following drugs prescribed on record during the first 12 weeks after delivery: anticholinergics: fesoterodine, flavoxate, imidafenacin, oxybutynin, propiverine, solifenacin, tolterodine, trospium, β_3_-adrenergic agonists, mirabegron, α-adrenergic agonists, pseudoephedrine, phenylephrine, serotonin-norepinephrine reuptake inhibitors, or duloxetine.

### 2.4. Covariates

In order to adjust for socioeconomic status, we assessed maternal age (15–49 years), income level (quintile, 1Q to 5Q), type of insurance (self-employed, employee insured, or medical aid), and residential area (rural and urban area). We also analyzed detailed obstetric information, particularly obstetric history and obstetric complications, as covariates. Obstetric history included mode of delivery (spontaneous vaginal, instrumental, or cesarean section delivery), parity (1st (nulliparous), 2nd, more than 3rd), twin birth status (yes or no), and maternal comorbidities. Maternal comorbidities were measured at the diagnosis codes during pregnancy, including pre-existing hypertension (O10), gestational hypertension, pre-eclampsia or eclampsia (O11, O13, O14, O15, O16), gestational diabetes mellitus (O24), abnormal findings or suspected fetal problems (O28, O35, O36), premature rupture of membranes (O42), placental disorders (O43), placenta previa (O44), and premature separation of the placenta (O45). Obstetric complications were assessed using the intrapartum diagnosis codes, including severe perineal laceration during delivery (O703, O704), other obstetric trauma (O70), postpartum hemorrhage (O72), puerperal sepsis (O85), infection of obstetric surgical wounds (O860), venous complications in the puerperium (O870, O871, O873, O878, O879), obstetric embolism (O88), complication of anesthesia during the puerperium (O89), puerperium (O90), and other maternal diseases (O98, O99). 

### 2.5. Statistical Analysis

This study used frequency and percentage to present the distribution of participants’ general characteristics. The associations between UI and PPD were analyzed using time-to-event methods. Cumulative incidence curves were generated for comparison of unadjusted PPD rates according to UI status. Cox proportional hazards models were used to calculate adjusted hazard ratios (HRs) and 95% confidence intervals (CIs) as an estimate of the relative rate of PPD. The proportionality assumption was tested by plotting Schoenfeld-like residuals. In addition, we assessed a combination of UI and mode of delivery factors with their associations to PPD using a Cox proportional hazards model. All statistical analyses were performed using SAS 9.4 (SAS Institute, Inc., Cary, NC, USA). The desired level of significance was set at *p* < 0.05.

## 3. Results

The general characteristics of the study population are shown in Table 1. The total population was 83,066, the incidence of UI during the first three months was 5393 (6.49%), and PPD was noted in 691 women (0.83%) during the first six months after delivery. The incidence of PPD was 691 (0.83%) in total.

The cumulative incidence of PPD according to UI is presented in Supplementary Figure S1. In women with UI, the incidence of PPD was significantly higher within three months after childbirth than in those without UI (*p* < 0.0001).

Table 2 shows the results of the association between risk factors and PPD using Cox proportional hazards analysis. The risk of PPD was 2.04 times higher in women with UI (HR 2.04, 95% CI: 1.63–2.57). In addition, the risk of PPD was significantly higher in women aged 15–19 years (HR 1.48, 95% CI 1.07–2.05), older than 40 years (HR 1.56, 95% CI 1.04–2.34, *p* = 0.0301), those with the lowest income level versus the highest income level (HR 1.34 95% CI 1.02–1.77), and in those with CS delivery versus SV delivery (HR 1.30, 95% CI 1.11–1.51, *p* = 0.001).

**Figure 1:**
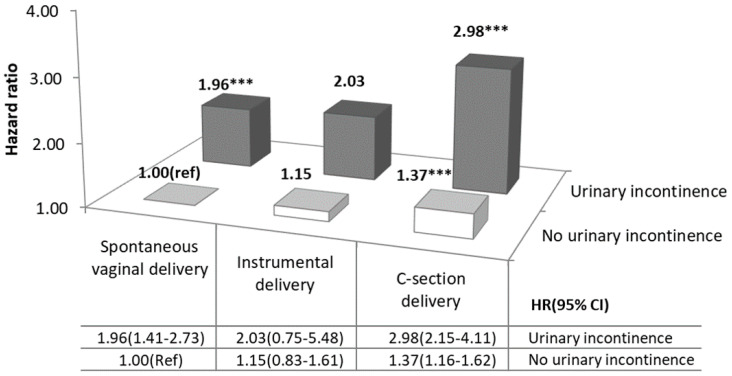
Combined effect of urinary incontinence and mode of delivery on postpartum depression within six months after childbirth. Adjusted for maternal age, income level, type of insurance, residential area, parity, twin birth status, maternal comorbidities, obstetric complications, and year. *** *p* < 0.001.

Figure 1 shows the results of the analysis of combinations of UI and mode of delivery on PPD using the Cox proportional hazards model. Women with UI who had SV or CS deliveries had a higher risk of PPD than those without UI who had SV (UI and SV: HR 1.96, 95% CI: 1.41–2.73; UI and CS: HR 2.98; 95% CI, 2.15–4.11). In addition, women without UI who had CS delivery had a higher risk of PPD than those in the reference group (HR 1.37, 95% CI 1.16–1.62).

## 4. Discussion

In this study, we found that UI was associated with an increased risk of PPD six months after childbirth in a large and long-term follow-up sample. In addition, women with CS deliveries had a significantly higher risk of PPD. 

Our results support previous prospective research that presented a relationship between UI and PPD. A prospective cohort study showed that women with UI were at a high risk of PPD six weeks after childbirth [5] and another revealed that women with UI were at a 2.23-times higher risk of PPD at six–seven months after childbirth than those without UI [16]. Another cross-sectional study also found an association between PPD and symptoms of urge incontinence [9]. However, some previous research found contrasting results. One study found that there was no association between depressive and urinary symptoms at one-year postpartum and that only overactive bladder syndrome was associated with depressive symptoms during pregnancy [14]. Another study reported that UI did not have a statistically significant association with childbirth and maternal depression in the first 12 months postpartum [20]. This relationship was still uncertain, probably because little attention has been paid to the distinctive influence of UI on PPD. Several studies have estimated the association between PPD and physical health after delivery, including UI [15,16], or assessed the effect of depression and UI during pregnancy [21]; however, they could not provide evidence of a direct effect of UI on PPD. In addition, the smaller sizes of the populations in previous studies contributed to reducing the statistical significance of the association between UI and PPD. By using diagnostic codes and prescriptions, we more comprehensibly determined the effect of UI on PPD six months after childbirth in a large population, which, to our knowledge, has not been attempted previously. 

In addition, those with CS deliveries were at an increased risk of PPD compared to those with SV deliveries. A previous study explained that women who delivered via CS might be at an increased risk of PPD because of a greater risk for pregnancy-related complications, whether intrapartum or postpartum [9]. Another study explained that CS deliveries might be related to feelings of failure, lack of control, and decreased self-esteem, resulting in a high risk for depression [22]. In contrast, other research has shown that UI is expected to be more common in women with SV deliveries than in those with CS because of a high risk of pelvic floor damage [9]. As a result, SV delivery is related to an increased risk of UI as a chronic disorder, which might also lead to PPD [14]. However, in this study, women with CS deliveries were at a higher risk of PPD than those with SV deliveries possibly because CS delivery might also induce adverse physiological outcomes, such as infection, bladder or surgical injuries, uterine rupture, and hysterectomy caused by postpartum hemorrhage, etc. [23]. These outcomes and surgical trauma might increase stress, which might affect psychological function and increase the risk of PPD [19]. Regarding combination effects, the results showed that both SV and CS delivery women with UI and those without UI who had CS delivery had a significantly higher risk of PPD than those without UI and having SV, which suggests that UI could be a risk factor for PPD in both SV and CS.

UI might affect PPD development through the following mechanism: Dysregulation caused by individual serotonergic mechanisms or through other neuropathways may potentially underlie the relationship between urge incontinence and depressive disorders as serotonergic activity affects regulating micturition by blocking the central pathways and improves bladder storage [9].

This study has several limitations. First, as our study assessed PPD using diagnostic codes, there was a potentially lower incidence of PPD as a result. While a previous study showed that PPD was prevalent in approximately 17% of postpartum women [24], our study found PPD to exist in less than 1% of the study population. Several Korean studies have shown that Korean women had a very low diagnosis rate of PPD six months after childbirth because of Koreans’ negative perceptions of mental disorders [3,6,25]. Additionally, PPD in our study did not reflect true maternal depression due to lack of awareness of mothers toward their depressive symptoms and appearance of symptom severity, as well as related unwillingness to seek help for PPD. Therefore, a selection bias, a non-response bias, or an erroneous classification by the women who misinterpreted the questionnaire might potentially exist in our study. Second, the rate of UI might have been underestimated because we identified UI only via diagnostic code and by prescription of UI drugs, excluding non-pharmacological therapy such as lifestyle interventions, pelvic floor muscle training, or bladder training. A previous study revealed that UI occurred in 5–36% of postpartum women [10]. In this study, the rate of UI three months after childbirth was 6.5%, which means that we might have missed cases of UI, although we could determine the exact time of UI occurrence using the prescription drug data. Third, while we tried to identify and adjust for potentially unmeasured variables, PPD in this study might have been affected by several other unmeasured confounders. These potential confounders include: maternal hormonal changes, depressive genetic factors, maternal BMI, birth weight, infant temperament, other physical health status, quality of life, marital relationship, self-esteem, childcare stressors, unplanned or unwanted pregnancy, marital status, and lack of social support [26]. 

Although the present study has some limitations, this study has several strengths. First, a nationwide cohort study with a population-based design was conducted using the NHIS-NSC data, which had a large sample size. Second, the recall bias problem was solved in this study by utilizing diagnostic codes and prescription records based on claims data compared to previous research based on self-reported questionnaires to assess PPD and UI. Third, we tried to consider various obstetric complications and comorbidities in order to adjust for case mix.

## 5. Conclusions

This study found that postpartum women with UI were at a higher risk for PPD compared with those without UI. Further research must determine which exact clinical and socioeconomic factors contribute to increasing the risk of UI and whether those factors are related to PPD. In addition, it would be necessary to determine the mediation effect of the relationship between UI and PPD by mode of delivery. Additionally, further research also should evaluate specific physical conditions, such as UI for mothers who display depressive symptoms. Therefore, policymakers should prioritize strategies that encourage the identification of mental health problems related to physical and emotional conditions and should promote the knowledge that UI may be a risk factor for PPD in maternity care.

## Figures and Tables

**Table 1:** General characteristics of the study population.

	Urinary Incontinence					
	No		Yes		Total	
	*n*	(%)	*n*	(%)	*n*	(%)
**Postpartum Depression**						
No	77,068	(99.22)	5307	(98.41)	82,375	(99.17)
Yes	605	(0.78)	86	(1.59)	691	(0.83)
**Maternal Age (Year)**						
15–24	3625	(4.67)	256	(4.75)	3881	(4.67)
25–29	23,428	(30.16)	1610	(29.85)	25,038	(30.14)
30–34	37,030	(47.67)	2592	(48.06)	39,622	(47.70)
35–39	11,950	(15.39)	837	(15.52)	12,787	(15.39)
≥40	1640	(2.11)	98	(1.82)	1738	(2.09)
**Income Level**						
1Q	7474	(9.62)	511	(9.48)	7985	(9.61)
2Q	11,177	(14.39)	820	(15.20)	11,997	(14.44)
3Q	20,151	(25.94)	1416	(26.26)	21,567	(25.96)
4Q	25,416	(32.72)	1703	(31.58)	27,119	(32.65)
5Q	13,455	(17.32)	943	(17.49)	14,398	(17.33)
**Type of Insurance**						
Self-employed insured	21,716	(27.96)	1628	(30.19)	23,344	(28.10)
Employee insured	55,693	(71.70)	3747	(69.48)	59,440	(71.56)
Medical aid	264	(0.34)	18	(0.33)	282	(0.34)
**Residential Area**						
Rural	22,883	(29.46)	1712	(31.74)	24,595	(29.61)
Urban	54,790	(70.54)	3681	(68.26)	58,471	(70.39)
**Mode of Delivery**						
Spontaneous vaginal delivery	43,947	(56.58)	3008	(55.78)	46,955	(56.53)
Instrumental delivery	4975	(6.41)	282	(5.23)	5257	(6.33)
Cesarean section delivery	28,751	(37.02)	2103	(38.99)	30,854	(37.14)
**Parity**						
1 (Nulliparous)	50,014	(64.39)	2949	(54.68)	52,963	(63.76)
2	24,458	(31.49)	2185	(40.52)	26,643	(32.07)
3+	3201	(4.12)	259	(4.80)	3460	(4.17)
**Twin Birth Status**						
Singleton	76,574	(98.59)	5314	(98.54)	81,888	(98.58)
Twin	1099	(1.41)	79	(1.46)	1178	(1.42)
**Maternal Comorbidities**						
0	50,164	(64.58)	3409	(63.21)	53,573	(64.49)
1+	27,509	(35.42)	1984	(36.79)	29,493	(35.51)
**Obstetrical Complications**						
0	65,904	(84.85)	4591	(85.13)	70,495	(84.87)
1+	11,769	(15.15)	802	(14.87)	12,571	(15.13)
	77,673	(93.51)	5393	(6.49)	83,066	(100.00)

**Table 2:** Cox proportional hazard ratios for postpartum depression within six months.

	Postpartum Depression			
	Person-Days	*n*	HR	95% CI
**Urinary Incontinence**				
No	13,928,597	605	1.00	
Yes	963,536	86	2.04	(1.63–2.57)
**Age (Year)**				
15–24	694,617	46	1.48	(1.07–2.05)
25–29	4,490,245	203	1.06	(0.88–1.27)
30–34	7,105,892	300	1.00	
35–39	2,290,843	115	1.13	(0.91–1.41)
≥40	310,536	27	1.81	(1.21–2.71)
**Income Level**				
1Q	1,429,083	94	1.34	(1.02–1.77)
2Q	2,149,874	102	0.99	(0.76–1.29)
3Q	3,867,094	161	0.90	(0.71–1.14)
4Q	4,864,234	215	0.98	(0.78–1.22)
5Q	2,581,848	119	1.00	
**Type of Insurance**				
Self-employed insured	4,184,131	208	1.06	(0.90–1.25)
Employee insured	10,657,715	477	1.00	
Medical aid	50,287	6	1.72	(0.75–3.94)
**Residential Area**				
Rural	4,407,834	210	1.00	(0.85–1.18)
Urban	10,484,299	481	1.00	
**Mode of Delivery**				
Spontaneous vaginal delivery	8,425,082	337	1.00	
Instrumental delivery	942,528	44	1.14	(0.83–1.57)
Cesarean section delivery	5,524,523	310	1.39	(1.19–1.63)
**Parity**				
1 (Nulliparous)	9,491,220	459	1.16	(0.98–1.39)
2	4,780,780	201	1.00	
3+	620,133	31	1.18	(0.80–1.74)
**Twin Birth Status**				
Singleton	14,680,583	685	1.99	(0.89–4.47)
Twin	211,550	6	1.00	
**Comorbidities During Pregnancy**				
0	9,605,024	435	1.00	
1+	5,287,109	256	1.08	(0.93–1.27)
**Obstetrical Complications**				
0	12,638,226	586	1.00	
1+	2,253,907	105	1.04	(0.85–1.28)

Adjusted for year.

## Data Availability

Data was obtained from the National Health Insurance Sharing Service and are available from at https://nhiss.nhis.or.kr/bd/ab/bdaba000eng.do with the permission of the National Health Insurance Service.

## Supplementary Materials

The following are available online at https://www.mdpi.com/1660-4601/18/2/437/s1, Figure S1: Cumulative incidence curves for the occurrence of postpartum depression with urinary incontinence within 12 weeks of delivery.
