# Supplementary material for: Does Urinary Incontinence and Mode of Delivery Affect Postpartum Depression? A Nationwide Population-Based Cohort Study in Korea
Source: Int J Environ Res Public Health. 2021 Jan 8;18(2):437. doi: 10.3390/ijerph18020437 (PMC7827536; doi:10.3390/ijerph18020437)

Supplementary file

Supplementary Figure 1. Cumulative incidence curves for the occurrence of postpartum depression with urinary incontinence within 12 weeks of delivery.

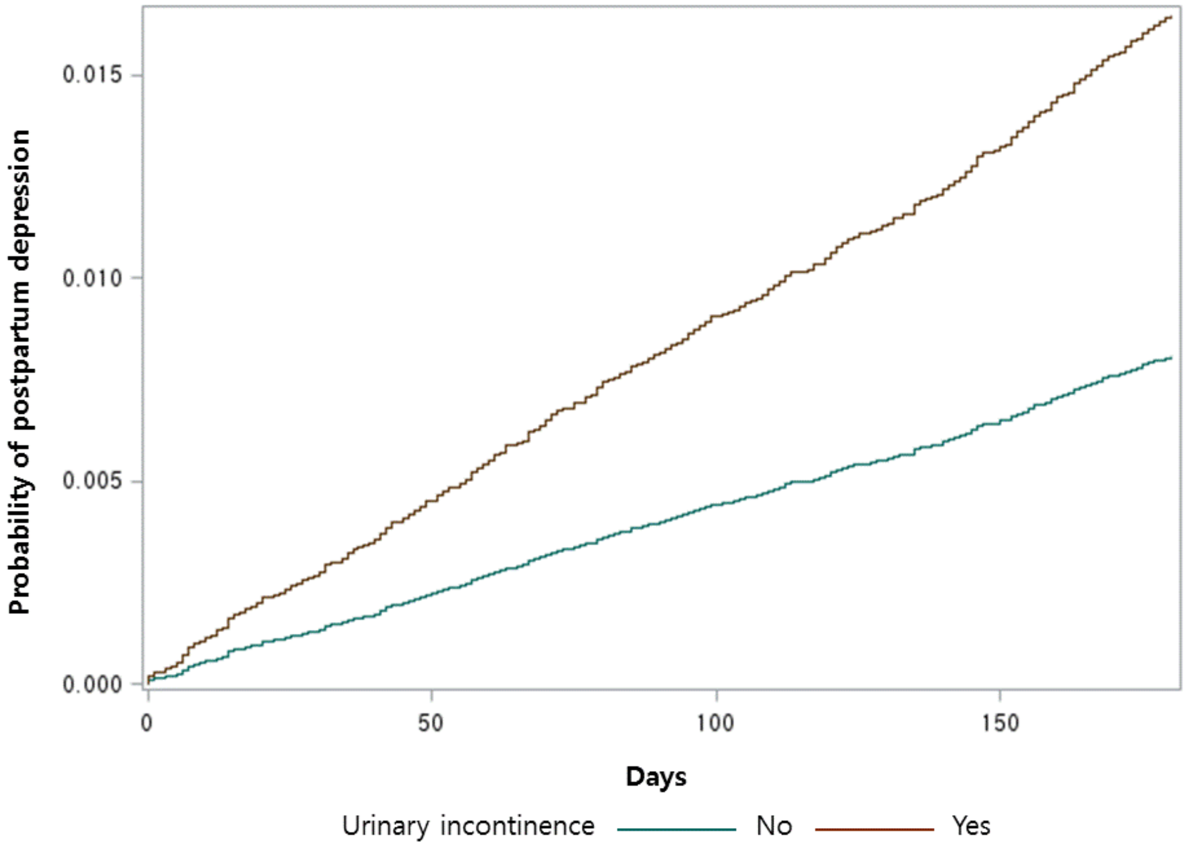

Supplement: Supplementary file 1 [file ijerph-18-00437-s001.pdf]
